# Histamine and Skin Barrier: Are Histamine Antagonists Useful for the Prevention or Treatment of Atopic Dermatitis?

**DOI:** 10.3390/jcm4040741

**Published:** 2015-04-21

**Authors:** Anna De Benedetto, Takeshi Yoshida, Sade Fridy, Joo-Eun S. Park, I.-Hsin Kuo, Lisa A. Beck

**Affiliations:** Department of Dermatology, University of Rochester Medical Center, Rochester, NY 14642, USA; E-Mails: takeshi_yoshida@urmc.rochester.edu (T.Y.); sade_fridy@urmc.rochester.edu (S.F.); joo-eun.s.park@rochester.edu (J.-E.S.P.); ihsinkuo@gmail.com (I.-H.K.); lisa_beck@urmc.rochester.edu (L.A.B.)

**Keywords:** atopic dermatitis, histamine, histamine receptors, skin barrier, tight junction

## Abstract

Atopic Dermatitis (AD), the most common chronic inflammatory skin disease, is characterized by an overactive immune response to a host of environmental allergens and dry, itchy skin. Over the past decade important discoveries have demonstrated that AD develops in part from genetic and/or acquired defects in the skin barrier. Histamine is an aminergic neurotransmitter involved in physiologic and pathologic processes such as pruritus, inflammation, and vascular leak. Enhanced histamine release has been observed in the skin of patients with AD and antihistamines are often prescribed for their sedating and anti-itch properties. Recent evidence suggests that histamine also inhibits the terminal differentiation of keratinocytes and impairs the skin barrier, raising the question whether histamine might play a role in AD barrier impairment. This, coupled with the notion that histamine’s effects mediated through the recently identified histamine receptor H4R, may be important in allergic inflammation, has renewed interest in this mediator in allergic diseases. In this paper we summarize the current knowledge on histamine and histamine receptor antagonists in AD and skin barrier function.

## 1. Atopic Dermatitis (AD) and the Skin Barrier

AD is the most common inflammatory skin disease, affecting up to 15 million Americans (about 17% of children and 6% of adults) [1]. Based on important discoveries over the last decade, it is thought that AD develops as a consequence of an acquired or genetic defect in the skin’s barrier [2,3]. The hypothesis is that these defects promote a more robust immune response to microbes, allergens, antigens, and irritants, and in the case of an atopic subject this response will lead to a largely T helper type 2 (Th2) cell infiltrate characterized by the release of interleukin (IL)-4 and IL-13. The interdependence of the immune and physical barrier systems is now a very active area of investigation and likely to lead to novel approaches to disease prevention and treatment.

It is widely accepted that the stratum corneum (SC) is dysfunctional in AD as a result of abnormal lipids (e.g., reduced ceramides and free fatty acids, an increase in unsaturated chain length) [4], altered expression of epidermal differentiation genes (e.g., loricrin, small proline-rich region proteins (SPRR)), *filaggrin* null-mutations, imbalance of proteases and protease inhibitors, and trauma from a chronic itch-scratch cycle (reviewed in [5]). We have focused most of our studies on a barrier structure found just below the SC, namely tight junction (TJ). In epithelial cells, TJs function as the “gate” for paracellular (*i.e.*, space between adjacent cells) passage of ions and solutes, which indirectly affects water transport (review in [6]). We have observed a TJ defect in the epidermis of AD subjects, as well as reduced expression of key TJ components, including claudin (cldn)-1 and -23 [7]. More recently, other studies have confirmed and expanded these findings by demonstrating reduced expression of other claudins (e.g., cldn-4 and 8) in the skin of subjects with AD [8,9].

The notion that an impaired skin barrier is a critical feature in the pathogenesis of AD presents an opportunity to develop new therapeutics aimed at repairing the skin barrier as an alternative or complementary approach to the more traditional anti-inflammatory therapies. Importantly, AD often precedes the development of other allergic diseases such as asthma and allergic rhinitis and this pattern of disease progression is often referred to as the Atopic March. This has led many to speculate that effective treatments for AD may diminish the risk for or severity of asthma. In 2002, annual US health care costs for AD were estimated to be as high as $3.8 billion, similar to emphysema and epilepsy [1]. More recently, a US population-based study showed that adult AD subjects have a significantly larger health burden with substantial out-of pocket costs, greater indirect costs (e.g., lost work days) and increased access to the health care system [10]. Despite its high prevalence, effects on quality-of-life and economic burden, there are still few effective treatments for AD and most have focused on inhibiting inflammation. Not surprisingly, this has resulted in high patient (and physician) dissatisfaction with AD management.

To date, AD treatments have exclusively targeted inflammation with the widespread use of topical corticosteroids and frequent use of systemic immunosuppressive drug (reviewed in [11]). As a potentially safer alternative to topical steroids, topical calcineurin antagonists (*i.e.*, tacroliums and pimecrolimus) were developed and received FDA approval in 2000 and 2001, respectively. In the USA, there are no systemic therapies approved by the FDA for treatment of AD. There are very few well-designed trials to support the off-label use of systemic immunosuppressives, with the possible exception of cyclosporine (reviewed in [12]). Cyclosporine is the only systemic treatment approved in some European countries for patients with severe AD [13]. Systemic steroids are often used in clinical practice to control flares. However, clinical studies to support their use in AD are surprisingly absent [14].

Recently a fully human monoclonal antibody directed against the IL-4 receptor α subunit (shared by the Type 1 and Type 2 IL-4 and IL-13 receptors, resulting in the blockage of both IL-4 and IL-13 signaling), called dupilumab (Regeneron Pharmaceuticals), has been shown to be highly effective for the treatment of moderate to severe adult AD subjects [15]. Dupilumab is currently poised to be the first systemic therapy to receive FDA approval for the treatment of adults with moderate to severe AD.

On the other hand, emollient therapy has been recognized for a long time as a critical component of AD patients’ management (reviewed in [11]). Recently it was shown that high-risk neonates who had emollient applied to the entire body on a daily basis since birth halved their risk for AD development [16]. Similarly, a study performed in Japan showed that daily use of emulsion-type moisturizer for the first 32 weeks of life significantly reduced the risk of developing AD [17].

Although the overall hypothesis is that emollients help restore the skin barrier function, mechanistic studies need to be done to test this theory.

Historically, histamine has been recognized as a potent inducer of pruritus. This, coupled with the increased histamine release observed in the skin of patients with AD [18], fostered the widespread use of Histamine Receptor 1 (H1R) antagonists in this disease. However, their clinical efficacy remains controversial. Several authors and clinicians have argued that antihistamines have a role in AD management largely for their soporific effects, essentially as a sleep aid (reviewed in [12]). A recent systematic review reported no high-level evidence to support or refute the efficacy of oral H1 antihistamines as monotherapy in AD patients [19]. Topical antihistamines have also been tried as treatment for AD. Studies evaluating the efficacy of topical doxepin (H1R/H2R and possibly H4R antagonist [20]) in the treatment of AD are conflicting [21,22]. Currently, topical antihistamines are not recommended because of their risk of absorption and contact dermatitis [11]. As we will discuss later in this review, the limited clinical efficacy of antihistamines might reflect their minimal effects on H4R-mediated actions. In addition, it should be highlighted that the vast majority of clinical trials were powered based on itch reduction as the primary endpoint. If one considers that a number of other mediators such as substance P, nerve growth factor, and IL-31 may also be mediating some (or all) of the pruritus observed in AD patients (reviewed in [23]) then it is not surprising that antihistamines have shown limited efficacy in mitigating itch.

In this review we will summarize what is known about histamine’s effects and the role of individual histamine receptors on epidermal skin barrier function. We will discuss how this information helps us better understand AD pathogenesis and the development of new therapeutic strategies.

## 2. Histamine Overview

Histamine (2-[4-imidazolyl]-ethylamine) is an aminergic neurotransmitter involved in numerous physiologic and pathologic processes, including pruritus, inflammation, and vascular leak. More than a century ago in 1910, Drs. Dale (recipient of the Nobel Prize for Medicine in 1936) and Laidlaw recognized that histamine has biological effects that mimic what is seen in an anaphylactic reaction [24]. A few years later histamine was isolated from lung and liver tissues and named histamine after the Greek word *histos* (tissue). In 1937, Drs. Bovet (recipient of the Nobel Prize in Physiology and Medicine in 1957) and Staub identified the first compounds capable of blocking histamine-mediated anaphylactic reactions [25]. Ever since, this has been an active and productive field of investigation, with a number of H1R and H2R blockers reaching the lofty blockbuster status defined as annual sales of ≥$1 billion. In fact, cimetidine (H2R-blocker; Tagamet^®^, GlaxoSmithKline, London, UK) was the first ever blockbuster drug (1985) [26].

Mast cells, basophils, and enterochromaffin cells (found in the gastric mucosa) are widely recognized cellular sources of histamine. However, other cells, including T cells and even keratinocytes, have been shown to produce histamine in response to stimulation [27,28]. The enzyme histidine decarboxylase (HDC) is responsible for histamine synthesis from the amino acid l-histidine. Of note, histamine can be also produced (from l-histidine via HDC) by some fermentative bacteria, including *Lactobacilli* in the gut [29,30]. This, coupled with recent knowledge about the potential role played by the skin microbiome in AD (reviewed in [31,32,33]), suggests a fascinating mechanism by which cutaneous bacteria might influence skin homeostasis.

In mast cells and basophils, histamine is stored in large quantities and quickly released upon stimulation. In other cell types, such as T cells and dendritic cells, histamine is newly synthesized and released after stimulation. HDC protein expression has recently been reported in cultured human keratinocytes and in the epithelial compartment of skin sections (by immunohistochemistry) [34]. Interestingly, *in vitro* studies using a human keratinocyte cell line (HaCat) demonstrated that HDC expression could be enhanced by stimulation with mediators present in AD skin lesions (*i.e.*, TNFα, thymic stromal lymphopoietin [TSLP], and house dust mite extract) and it was associated with greater histamine release [34]. These authors also reported greater HDC intensity staining in the epidermis of AD subjects [34].

Histamine concentrations measured in various tissues range from 10^−5^ to 10^−3^ M [35]. Unfortunately, the methods for measuring histamine in plasma/serum or tissue samples are not very reliable or reproducible. Gutzmer *et al.* recently summarized published studies reporting histamine concentrations in different inflammatory skin diseases, including AD (see Table 1 in [36]). Authors highlighted the different methods of detection used and the variability in histamine concentrations measured in healthy and disease states and concluded that there was a need for new detection methods. A new method using liquid chromatography tandem mass spectrometry to measure histamine in plasma and tissues has recently been reported [37].

Histamine can bind to four receptors belonging to the large family of rhodopsin-like G-protein-couples receptors (GPCRs), named in chronological order based on their discovery as H1R, H2R, H3R, and H4R, only described in 2000 [38,39,40,41]. The biological effects of histamine stimulation are determined by the activation of one (or more) of the histamine receptors [42]. Several cell types, including epithelial and endothelial cells, dendritic cells, and neutrophils as well as T and B lymphocytes express both H1R and H2R [36,43]. H3R expression is localized primarily in the central nervous system. H4R is expressed by bone-marrow-derived cells, including T lymphocytes, dendritic cells, mast cells, and eosinophils as well as epithelial cells [44,45,46,47,48]. Interestingly, it has been shown that Langerhans cells, which are a subset of professional antigen-presenting cells that reside in the epidermis, selectively express H4R but not H1R or H2R [49,50]. Human keratinocytes express H1R, H2R, and H4R [51]. This is in contrast with murine keratinocytes where H1R, but not H4R, is expressed constitutively. However, it was shown that H4R expression could be induced upon innate immune stimulation with LPS and peptidoglycan [51]. This difference in H4R expression between human and mouse keratinocytes should be taken into consideration when performing studies investigating histamine biology in murine models of human disease.

The histamine-induced signaling cascade is quite complex. We will superficially summarize this topic and refer readers to expert reviews on this topic for greater details [42,52,53,54]. It is a widely held belief that most of the allergic and inflammatory actions of histamine are mediated by the H1R, a G_αq/11_ receptor. Activation of the cellular process after histamine binding to H1R occurs via phospholipase C-mediated calcium mobilization, protein kinase C activation, and nuclear factor-κB-mediated signaling pathways (reviewed in [54]). A hallmark of the H2R signaling pathway is the formation of cAMP [55], while H4R signals through G_i/o_ receptors, resulting in inhibition of forskolin-induced cAMP production, intracellular calcium mobilization, and actin polymerization [40,55,56]. In addition, H4R has been reported to activate mitogen-activated protein kinases [57] and the JAK/STAT signaling pathway [58,59]. Importantly, histamine receptors can form dimers and oligomers, which allow interaction among histamine receptors as well as other G protein-coupled receptors, and this further increases the complexities of downstream signaling events in response to histamine stimulation.

Several commercially available antihistamines block either H1R or H2R or both, while H3R and H4R are currently being tested in clinical trials [60,61]. Based on their good safety profile, these drugs have been largely used for symptomatic treatment of allergic diseases, pruritic conditions, and gastroesophageal reflux disease (GERD) [62,63]. Antihistamines are recognized for a number of anti-inflammatory effects including inhibitory effects on mast cell and basophil degranulation, inhibition of adhesion molecules and eosinophil or neutrophil chemotaxis, enhancing apoptosis of inflammatory cells, reducing neuroinflammation, and cytokine/chemokine expression [64]. More recently it has become clear that some of the FDA-approved antihistamines are not as selective as we once thought and some have binding affinities for other HR receptors, albeit typically at doses not achieved with standard clinical dosing [64]. Importantly, none of the FDA-approved antihistamines antagonize H3R or H4R at standard dosing regimens. In an epicutaneous allergen challenge murine AD model, treatment with the selective H1R antihistamine, olopatadine, not only suppressed inflammation and scratching by inhibiting cytokine/chemokine production (e.g., IL-31, TSLP, TARC) but also improved the skin barrier function [65,66,67,68,69]. Olopatadine has inhibitory effects on the release of inflammatory mediators (e.g., histamine, leukotriene, thromboxane, and tachykinins), which could explain these broad anti-allergic properties [70]. Olopatadine was approved in 2000 in Japan for the treatment of several conditions including AD, chronic urticaria, and allergic rhinitis. However, in the USA and the European Union it is only available as a topical preparation for ophthalmic or nasal administration.

Relatively selective H3R and H4R blockers are currently in various stages of development by many pharma/biotech companies. Since H4R was identified at the beginning of this century, there have been a tremendous number of publications and patent applications. Preclinical data have highlighted the immunomodulatory properties of H4R, including effects on the chemotaxis of eosinophils [48] and mast cells [71], accumulation of FoxP3+ T cells [72] as well as modulation of inflammatory mediators (e.g., downregulation of IL-12 and CCL2) produced by monocytes [73,74]. This has increased enthusiasm that H4R (alone or in association with H1R antagonist) may be an effective new drug class for the treatment of allergic diseases [75,76]. High-throughput drug screening has led to the identification of new selective non-imidazole H4R ligands. As a result, several compounds are currently in preclinical and early clinical development. In Japan, a Phase 2a randomized, double-blind, placebo-controlled, multicenter, parallel-group clinical trial has tested the novel H4R antagonist, JNJ-39758979, in adult subjects with moderate AD. The study was terminated in response to two cases of neutropenia in the treatment group. Although the safety profile of this H4R antagonist remains a real concern, some beneficial effects were observed on disease severity and itch scores [77]. More studies are definitely needed to clarify the safety and efficacy of this compound or other H4R antagonists in the clinical setting.

## 3. Histamine and the Skin Barrier

In addition to its pro-inflammatory and itch effects, there is a growing body of evidence demonstrating that histamine also plays a role in epidermal terminal differentiation and skin barrier function. Gschwandtner *et al.* [78] recently demonstrated that histamine dose-dependently suppressed epidermal differentiation as indicated by significant reductions in filaggrin, loricrin, and keratin10 expression using both cultured primary human keratinocytes and an epidermal skin model (e.g., raft culture). In the raft culture system they observed that treatment with histamine induced thinning of the epidermis (50%), which was especially notable for the stratum granulosum, and that these effects were mediated by H1R [78]. These observations are consistent with previous studies showing that treatment with histamine or receptor (H1R and H2R) agonists or antagonists modulates epidermal barrier recovery in murine models [79,80,81]. Topical application of histamine or dimaprit (H2R agonist) delayed skin barrier recovery after tape stripping, as measured by transepidermal water loss (TEWL) in hairless mice [80]. In contrast, treatment with histamine receptor antagonists (e.g., olopatadine/H1R, diphenhydramine/H1R, and cimetidine/H2R) improved epidermal barrier recovery [79,80,81].

It has been known for some time that histamine, as well other amines such as thrombin, disrupt tight junction (TJ) in endothelial as well as epithelial cells [82,83]. In airway epithelial cells, histamine reduced the expression of a key TJ molecule, ZO-1, and this effect was at least partially abrogated by pretreatment with mepyriamine (an H1R antagonist), but not by ranitidine (an H2R antagonist) [84]. In the human skin equivalent model, Gschwandtner *et al.* demonstrated a strong suppression of intercellular junction proteins, including TJ components (e.g., cldn-1, -4 and occludin) and desmosomal components (e.g., corneodesmosin and desmoglein-1) after treatment with histamine [78]. These abnormalities were also associated with enhanced penetration of biotin in their skin equivalent model [78]. In our laboratory, we have started to investigate the effect of histamine and selected histamine receptor (H1R, H2R, and H4R) antagonists on epidermal TJ function and composition. We have employed two complementary epidermal models: an *in vitro* system utilizing cultured primary human keratinocytes (PHK) and an *ex vivo* system with full-thickness epidermal explants isolated from discarded skin samples from elective surgeries. TJ integrity was quantified by measuring transepithelial electric resistance (TEER) and paracellular flux, as we have previously described [85]. Briefly, TEER was measured using an EVOMX voltohmmeter (World Precision Instruments, Sarasota, FL, USA). To evaluate the paracellular flux of PHK, 0.02% Fluorescein Sodium (Fluka, St. Louis, MO, USA) in PBS (Invitrogen/Gibco, Grand Island, NY, USA) was added to the upper chamber (apical side) and samples were collected from the lower chamber (basal side) after 30 min incubation for PHK culture. PHK, isolated from neonatal foreskin, were cultured in Keratinocyte-SFM (Invitrogen/Gibco, Grand Island, NY, USA) containing 5 ng/mL recombinant EGF, 50 µg/mL BPE, 1% Pen/Strep, and 0.2% Amphotericin B (Invitrogen/Gibco). To induce terminal differentiation, sub-confluent PHK were differentiated in DMEM (Invitrogen/Gibco) containing high calcium (1.8 mM) but no serum or growth factors. In previous studies we determined that PHK grown under these culture conditions are highly differentiated [85]. Histamine dihydrochloride (1–100 µM, Sigma-Aldrich, St. Louis, MO, USA) was added to the culture media from the time of differentiation and replaced every 48 h with the medium change. This model allows us to study the effect of histamine on TJ assembly. De-identified human discarded skin was obtained from the Pathology Department of the University of Rochester Medical Center with the approval of the institutional Research Subject Review Board. As we previously described [85], the epidermis was enzymatically separated from the dermis using dispase and the epidermal sheet was sandwiched between two sterile custom-made Plexiglas discs with an opening of 3-mm diameter and placed in modified Snapwell™ chambers (Corning; Corning, NY, USA). Fresh media (DMEM) with histamine (10 or 100 uM) or media alone were then added to both sides of the transwell. The TEER and paracellular flux of fluorescein of skin explants were measured at 24 h. This model allowed us to investigate the effect of histamine on already formed TJ.

**Figure 1:**
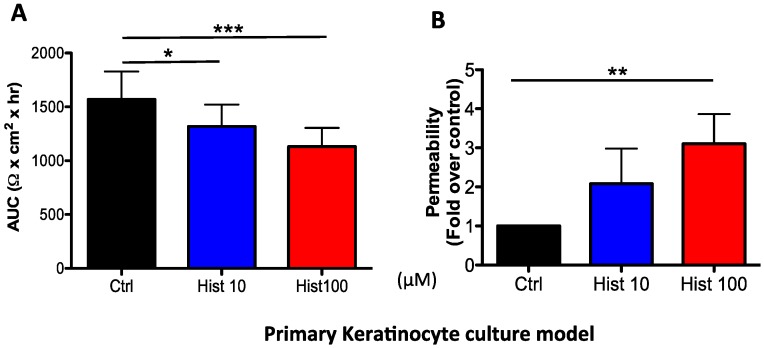
Histamine reduces TJ integrity in cultured primary human keratinocytes. Histamine dose-dependently reduced (**A**) transepithelial electric resistance (TEER) and (**B**) enhanced permeability to fluorescein. TEER is shown as mean area under the curve (AUC) ± SEM on *n* = 9 experiments; Permeability is shown as mean fold of control ± SEM of *n* = 16–10. Samples from the same donor were compared and a paired *t*-test was used for statistical analysis: * *p* < 0.05; ** *p* < 0.001, *** *p* < 0.0001.

**Figure 2:**
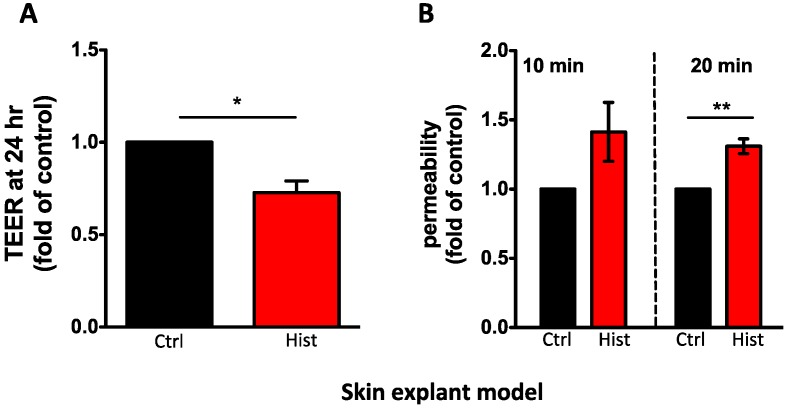
In an *ex-vivo* model evaluating full-thickness human epidermis placed in a modified micro-snapwell system, histamine (100 μM) (**A**) reduced TEER (0.7-fold) and (**B**) enhanced fluorescein permeability flux (20 min time point; 1.3-fold). TEER is shown as mean fold of control ± SEM on *n* = 3; permeability is shown as mean fold of control ± SEM of *n* = 3. Samples from the same donor were compared and a paired t-test was used for statistical analysis: * *p* < 0.05; ** *p* < 0.001, *** *p* < 0.0001.

In agreement with published data, we found that histamine reduced TJ integrity. In cultured PHK, we observed a dose-dependent reduction of TEER (10 and 100 μM, *p* < 0.001, *n* = 9; Figure 1A) and enhanced permeability (100 μM, *p* < 0.001, *n* = 16; Figure 1B). Using the *ex vivo* model, we confirmed that histamine (100 μM) also reduced TEER (0.7 fold, *p* < 0.05, *n* = 3; Figure 2A) and enhanced fluorescein permeability flux (1.3 fold, *p* < 0.05, *n* = 3; Figure 2B) in epidermal explants. Based on these findings, we concluded that histamine impaired TJ integrity. Studies are ongoing to establish the signaling pathways mediating the histamine-induced modulation of TJ. Based on published studies in other epithelial and endothelial cell models, we speculate that histamine may act directly on TJ composition; however it is possible that the effect on TJ is indirect and therefore secondary to histamines’ actions on other biological pathways. As mentioned earlier, it has been shown that histamine treatment prevents terminal differentiation, which is mediated by H1R [78]. Impairment of differentiation could potentially affect the development of a mature TJ network, which is typically observed at the level of the stratum granulosum. Additionally, Glatzer *et al.* [51] demonstrated that histamine induced keratinocyte proliferation that was mediated by H4R. Increased proliferation would likely reduce differentiation and TJ assembly. On the other hand, histamine has been shown to induce the production of a number of inflammatory mediators (e.g., IL-31, human β-defensin 2) [86], which could affect the skin barrier [87,88].

In summary, our preliminary data and published studies demonstrate that histamine disrupts epidermal barriers (TJ and stratum corneum) and that blocking histamine may prevent this unwanted action on the epidermal barriers. Human clinical trials evaluating the effects of specific HR antagonists on skin barrier function and inflammation are needed to better understand the best clinical use of old as well as new, more selective, antihistamines in the management of AD.

## 4. Conclusions

There is an ongoing debate about the notion that an impaired skin barrier promotes the development of AD. Whether this breach is the consequence of genetic mutations (*i.e.*, FLG null mutations) or something that develops in response to changes in the micro- or macro-environment or both is still unclear. An argument for the acquired pathway is the fact that a number of T-cell-derived cytokines (e.g., IL-4, IL-13, IL-25, IL-22, or IL-17A) found in AD skin can inhibit the epidermal expression of key barrier proteins such as filaggrin, loricrin, and involucrin, which are also markers of terminal differentiation [88,89,90,91]. As discussed in this manuscript, histamine can be added to this list of barrier-modulating agents. Histamine can affect skin barrier integrity by promoting proliferation and inhibiting differentiation of keratinocytes, by disrupting TJ integrity, or by indirectly modulating the parenchymal immune response. This knowledge, along with the observation that H1R or H4R blockade by itself may limit histamine-induced barrier disruption, has led to renewed interest in the role of histamine in allergic inflammation, and may lead to using blockades targeting H1R and H4R as “novel” prevention and/or treatment options for AD patients.
